# Editorial: Past, present and future contributions from the social cognitive theory (Albert Bandura)

**DOI:** 10.3389/fpsyg.2023.1258249

**Published:** 2023-08-07

**Authors:** Jesús de la Fuente, Douglas F. Kauffman, Evely Boruchovitch

**Affiliations:** ^1^School Education and Psychology, University of Navarra, Pamplona, Spain; ^2^School of Clinical Medicine, Medical University of the Americas-Nevis, Devens, MA, United States; ^3^School of Education, UNICAMP State University of Campinas, São Paulo, Brazil

**Keywords:** social cognitive theory, Albert Bandura, in memoriam Albert Bandura, present and future, self-regulation, internal- vs. external-regulation behavior theory

Cognitive Social Learning theory (Bandura, 1986) tries to understand how the acquisition of knowledge, beliefs, attitudes, and ways of thinking of the person with respect to the social environment occurs. The premise underlying this theory is that learning is a cognitive process that cannot be separated from the context in which it occurs, be it family, school or of any other nature. Albert Bandura was a giant in the field, with work that influenced social, cognitive, developmental, educational, and clinical psychology. His death on July 21, 2021 left a void in the filed of psychology. He will definitely be greatly missed. This Research Topic has been developed to pay tribute to him, from the aforementioned disciplines.

A total of 9 articles and 68 authors have contributed to the objective of showing recent models and evidence, derived from Albert Bandura's original theoretical model. Two papers, carried out by the participating researchers, analyze the effect of a key construct in Bandura: the Self-Efficacy. The first work is focused on the effect of *Self-efficacy beliefs as a predictor of quality of life and burnout among university lecturers* (da Mota et al.). The second analyzes *How does teacher-perceived principal leadership affect teacher self-efficacy between different teaching experiences through collaboration in China* (Xie et al.).

Other works have presented evidence regarding the effect of motivation in the teaching-learning process. The first refers to the *Predictive model of the dropout intention of Chilean university students* (López-Angulo et al.); the second, entitled *You and Me Versus the Rest of the World: The Effects of Affiliative Motivation and Ingroup Partner Status on Social Tuning* (Skorinko et al.).

An article has addressed another essential aspect of his theory, referred to the moral impact of disengagement, as an explanatory mechanism of aggression and antisocial behavior. The work entitled *The effect of individual and classroom moral disengagement on antisocial behaviors in Colombian adolescents* (Gómez-Plata et al.) shows this phenomenon.

An experimental work is focused on *Self-Regulated Learning*, an essential construction derived from A. Bandura's theory. Shows the *Short and Long-Term Effects on Academic Performance of a School-Based Training in Self-Regulation Learning* (Tuero et al.).

Finally, two papers show a new, broader model of self-regulation, derived from the Theory of A. Bandura, presenting types of *internal and external regulation*, applicable to different psychological contexts, in the paper entitled *Advances on Self-Regulation Models: A New Research Agenda Through the SR vs ER Behavior Theory in Different Psychology Contexts* [de la Fuente et al. (a); de la Fuente et al. (b)]. Complementarily, an initial validation study of the Assessment Scales of the Regulation/non-regulation/dyregulation construct (personal and contextual) is presented, in the work titled *Self- vs. External-Regulation Behavior Scale TM in different psychological contexts: A validation study* (de la Fuente, Pachón-Basallo, et al.).

In conclusion, this Research Topic is also dedicated to the incredible person and psychologist Albert E. Bandura (1925-2021). Dr. Albert Bandura, was one of the most influential psychologists of all time. Bandura pioneered the field of social learning theory (now called social cognitive theory) with his landmark Bobo doll experiment. He defined the construct of self-efficacy and proposed an agentic theory of human behavior that challenged the central tenants of behaviorism. Born in Alberta, Canada, in 1925, Bandura earned his undergraduate degree from the University of British Columbia in Vancouver and his graduate degree from the University of Iowa. He joined the faculty at Stanford University in 1953, where he served as the David Staff Jordan Professor of Social Science in Psychology. Bandura was elected APA president in 1973 and encouraged our organization to pursue matters of public interest. Bandura's significant contributions to the field of psychology were recognized in 1980 with APA's Distinguished Scientific Contribution Award and in 2004 with our Award for Outstanding Lifetime Contribution to Psychology. He also received the Gold Medal Award for Distinguished Lifetime Contribution to Psychological Science from APF and the Lifetime Career Award from the International Union of Psychological Science. In 2016, he was awarded the National Medal of Science by President Barack Obama.

## Author contributions

All authors listed have made a substantial, direct, and intellectual contribution to the work and approved it for publication.

## References

[B1] Bandura A. (1986). Social Foundations of Thought and Action. Upper Saddle River, NJ: Prentice Hall.

